# Tapping on a target: dealing with uncertainty about its position and motion

**DOI:** 10.1007/s00221-022-06503-7

**Published:** 2022-11-12

**Authors:** Eli Brenner, Cristina de la Malla, Jeroen B. J. Smeets

**Affiliations:** 1 Department of Human Movement Sciences, Vrije Universiteit Amsterdam, Van der Boechorststraat 7, 1081BT Amsterdam, The Netherlands; 2 Vision and Control of Action Group, Department of Cognition, Development, and Psychology of Education, Institut de Neurociències, Universitat de Barcelona, Barcelona, Spain

**Keywords:** Continuous control, Online control, Feedback, Arm movements, Vision, Human

## Abstract

Reaching movements are guided by estimates of the target object’s location. Since the precision of instantaneous estimates is limited, one might accumulate visual information over time. However, if the object is not stationary, accumulating information can bias the estimate. How do people deal with this trade-off between improving precision and reducing the bias? To find out, we asked participants to tap on targets. The targets were stationary or moving, with jitter added to their positions. By analysing the response to the jitter, we show that people continuously use the latest available information about the target’s position. When the target is moving, they combine this instantaneous target position with an extrapolation based on the target’s average velocity during the last several hundred milliseconds. This strategy leads to a bias if the target’s velocity changes systematically. Having people tap on accelerating targets showed that the bias that results from ignoring systematic changes in velocity is removed by compensating for endpoint errors if such errors are consistent across trials. We conclude that combining simple continuous updating of visual information with the low-pass filter characteristics of muscles, and adjusting movements to compensate for errors made in previous trials, leads to the precise and accurate human goal-directed movements.

## Introduction

Trying to understand human movements can be approached in a wide variety of ways. At one extreme is the study of neuromechanical issues, such as how muscle contractions and spinal reflexes bring about a desired posture (Feldman et al. 2007; Latash, 2010; Polit and Bizzi 1978), or other aspects of how movements can best be controlled (Scott 2004; Yeo et al. 2016). At the other extreme is the study of sensory issues, such as how directly coupling one’s movements to certain sensory information (Lee et al. 1999, 2001) or moving in a way that gives rise to certain sensory feedback (Chapman 1968) could guide one to one’s goal. Presumably, common movements are well adapted to their purpose as a result of the solution space having been explored extensively (Scholz and Schöner 1999; Scholz et al. 2000; Rosenbaum et al. 1995). The way movements are controlled, therefore, probably reflects a compromise between optimizing a combination of relevant factors (Kistemaker et al. 2014; Liu and Todorov 2007; Rosenbaum et al. 2001). The best solution may be different when one wants to maximize precision (Harris and Wolpert 1998) than when one wants to minimize energetic cost (Kuo 2007; Ren et al. 2007). It also probably depends on whether the whole path is relevant (as in writing or dancing) or mainly the endpoint (as in typing or grasping). The most effective approach to understanding why people move in a certain manner might, therefore, be different for different tasks.

Most studies on how movements are controlled assume that the sensory information that one relies on is correct. This is not a justified assumption considering that the precision of sensory estimates is obviously limited by the resolution of the sensory organs, and that judgments of attributes such as position (Kuling et al. 2016; Smeets et al. 2006), shape (Scarfe and Hibbard 2011) and motion (Stocker and Simoncelli 2006; Welchman et al. 2008) can be biased. In some tasks, such as intercepting moving targets, performance is even primarily limited by the extent to which people can make precise and unbiased sensory judgments (Brenner and Smeets 2015; Nelson et al. 2019), rather than by their ability to correctly execute a planned movement. It might, therefore, be worthwhile improving precision by accumulating information across time (Zimmermann et al. 2013). Moreover, people learn to avoid biases by compensating for perceived errors on previous attempts (Brenner and Smeets 2011; Körding and Wolpert 2004).

An attribute that is obviously important for any movement towards an object is the object’s position. Many daily-life interactions are with static objects. If it is evident that an object’s position is not changing, one might expect information about the position to be accumulated over tens or even hundreds of milliseconds. People gradually accumulate information about a target’s position when the circumstances are specifically designed to make it necessary to do so (Battaglia and Schrater 2007). However, normally a target’s position can be judged without combining information obtained at different times, and the position that is needed to guide an action is ultimately the position with respect to the actor’s body, rather than the position with respect to the environment. Thus, even when the target object is static with respect to the environment, the relevant position changes whenever the actor moves. This means that the actor’s movements need to be considered if one is to reliably accumulate information (Wolpert and Ghahramani 2000).

When interacting with moving objects, simply averaging position information is obviously not a good way to accumulate sensory information, even if one does consider one’s own movements. One way to solve this is to consider the object’s motion as well. One could anticipate that the object’s position at a future moment of interest follows from its current position and velocity, and accumulate information about this future position by recursively combining the previously predicted position with new predictions based on instantaneous sensory signals. One might even choose the weights for the prediction and the sensory signals that maximize the precision of the combined estimate (Kalman 1960). This requires some knowledge of the components’ individual precisions. The precision of the sensory information could be obtained from the sensory signals themselves (Ma et al. 2006). The precision of the prediction could be judged from recent experience (Narain et al. 2013). However, if the moving object’s position changes in a way that is not captured by the considered kinematics, for instance because forces accelerate or decelerate the object, accumulating information to optimize precision will introduce systematic errors.

What would happen if one would always rely on the newest sensory information rather than accumulating sensory information to improve one’s judgments? Constantly using the latest information to adjust the movement does not mean that the movement path will be as variable as the instantaneous sensory estimates, because the sensory information is used to direct the activation of the muscles that generate forces to accelerate the hand. In this sense, guiding movements will behave somewhat like a servo control that uses the current sensory information on position and velocity as input (a mass-spring damper system; McIntyre and Bizzi 1993; de Lussanet et al. 2002; Smeets and Brenner 1995a). The mechanical damping will smooth out the fastest fluctuations in the sensory information, so the movement will effectively be guided by a slightly smoothed version of the sensory information without independently accumulating sensory information.

How can we find out to what extent humans accumulate information to guide their movements? We approach this by analysing the responses to small, unpredictable target displacements at various instants during a goal-directed movement. If people always rely on the latest information, they should respond more vigorously to target displacements that occur later in the movement because the movement has to be adjusted in less time (Brenner et al. 2022; Oostwoud Wijdenes et al. 2011). If information is accumulated to increase precision, the vigour of the response might not increase in this systematic manner, because the rate at which new information influences the judged target position might decrease as more information is accumulated. We know that movements are adjusted to isolated target displacements (reviewed in Smeets et al. 2016). Here, we introduce many small displacements throughout a movement to examine how the response changes during the movement (as has been done to analyse responses to mechanical perturbations in the past; Lacquaniti et al. 1982; Soechting et al. 1981). In our first experiment, we investigate the extent to which participants’ fingers follow the position of the jittering target. In the four subsequent experiments, we examine how people use judgments of velocity to extrapolate the path of a moving target and how they deal with accelerating targets.

### Experiment 1: position

We examined the extent to which movements are adjusted to changes in target position at various times during a goal-directed action by asking participants to tap on a target that was jittering slightly in the lateral direction. The target was a 1.0 cm radius white disk. It was presented on a large screen at a frame-rate of 120 Hz. Between consecutive frames (i.e., every 8.3 ms) the target stepped 1.67 mm (1/6th of the target radius) leftward or rightward, with the direction of the step being chosen at random. This jitter was clearly visible to the participants. Given that it took participants about 500 ms to tap the target (see “Results”), the target changed position about 60 times during each trial.

### Methods

#### Participants and equipment

**Fig. 1:**
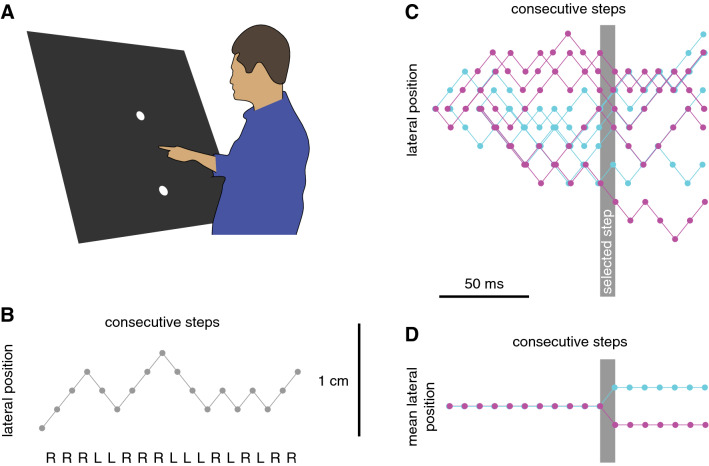
Methods of Experiment 1. **A** Participants stood in front of a large screen and moved their finger upwards from a starting point to a target to tap on it. The target appeared at a fixed position on the screen, but jittered laterally from then on. **B** Between the consecutive frames of each trial, the target always stepped slightly to the left (L) or to the right (R). **C** To analyse the responses, the trials were aligned at the moment of the tap and sorted into two sets (indicated by their colour) on the basis of the direction of the step at a certain time before the tap (the step in question is indicated by the grey bar). **D** When averaging across many trials, the only systematic difference between the two sets is the direction of the step at the selected time, so the sets differ in average target position by 3.33 mm from that moment onwards

Fifteen adults took part in the experiment, including one of the authors. The experiments were conducted in accordance with approval by the *Vaste Commissie Wetenschap en Ethiek van de Faculteit der Gedrags- en Bewegingswetenschappen*. This included having each participant sign an informed consent form. During the experiment, the participant was standing in front of a large screen (Techplex, 1.25 × 1 m; slanted 30° backwards; Fig. 1A). Images were projected onto this screen from behind (In-Focus DepthQ Projector). A 1.67 mm step size and 120 Hz frame rate corresponds with a velocity of 20 cm/s. The resolution was 800 × 600 pixels, so the step size was only about one pixel, but as the target was a disk the location of its centre was defined with sub-pixel precision.

An infrared marker was attached to the nail of the index finger of the participant’s preferred hand. This marker’s position was tracked at 500 Hz using an Optotrak 3020 system. The relationship between the marker’s position and the position of the tip of the finger with respect to items on the screen was determined before each session using a simple four-point calibration. In order to synchronize the kinematic data with the timing of the stimulus, a second marker was attached to the side of the screen. This marker briefly stopped emitting infrared light when light hit a light-sensor at the top left corner of the screen. We presented a flash at this corner of the screen at the moment the target first appeared. Since the Optotrak system uses the emitted infrared light to track markers, data for the second marker were missing at this moment, which allowed us to synchronize finger movements with the moment of target appearance to within 2 ms.

#### Procedure

There were 500 trials per participant. Each trial started when a participant placed his or her finger on a 1 cm radius, white starting point that was located 15 cm below the centre of the dark screen (when mentioning distances above and below the screen centre we are actually referring to distances along the slanted screen). Between 600 and 1200 ms later, the target appeared 10 cm above the screen centre (25 cm from the starting point). Participants then had another 700 ms to tap on the target. The moment of the tap was determined during the trial. A tap was considered to have occurred if the finger marker’s acceleration exceeded a threshold of 50 m/s^2^ away from the screen (the acceleration caused by the impact), while the finger was initially moving towards the screen and was less than 5 mm from the screen. The acceleration was determined on-line by taking the difference between the two displacements within three consecutive Optotrak measurements.

If participants did not tap on the screen within 700 ms of the target appearing, the target disappeared. If they tapped within 700 ms of the target appearing and the tip of their finger (as determined during the calibration) was within the target at the moment of the tap, the target remained where it had been at the time of the tap for 500 ms (without jittering) and the participant heard a sound indicating that the target had been hit. If they tapped in time but missed the target, the target moved away from the tapped position at 1 m/s. In both cases we considered the target to have remained where it had been presented until it was presented elsewhere. Participants could rest at any time by not placing their finger at the starting point. If they moved their finger away from the starting point before the target appeared, the target did not appear and they had to move their finger back to the starting point to wait for another 600 to 1200 ms before the target appeared.

#### Analysis

We excluded trials in which the screen was tapped after the target had disappeared. We also excluded trials in which the finger’s position was invisible for longer than 20 ms during the movement. Missing data for shorter durations were interpolated. We used a second-order Savitzky–Golay filter to obtain slightly smoothed estimates of the finger’s lateral position and velocity for each measured sample (Savitzky and Golay 1964). We fit a second order polynomial to the 9 position samples from 8 ms before to 8 ms after each sample and used the value of this polynomial at the moment of the sample in question as our measure of the position at that moment, and the value of its derivative at that moment as our estimate of the velocity at that moment.

As already mentioned, the target stepped 1.67 mm every 8.3 ms. Whether it stepped to the left or to the right was chosen at random on each frame, making the target jitter laterally, following a random walk (Fig. 1B). In order to determine how the way participants responded to such jitter depended on the timing of the step, we first aligned the trials at the moment of the tap. After doing so, we determined the responses to steps at each time from 400 ms before the tap until the moment of the tap. For each time, we sorted all of each participant’s trials on the basis of whether the step at that time was to the left or to the right (Fig. 1C). Sorting the trials in this manner gave us two sets of trials that differed in the direction of the step at that moment. The two sets of trials had about equal numbers of leftward and rightward steps at all other times. Consequently, from the time of the selected step onwards, the average position of the target differed between the two sets by 3.33 mm, twice the step size (Fig. 1D).

For each participant and moment of the step (relative to the tap), we sorted all movements according to the direction of the step (as described above) and determined how the finger’s average lateral position and velocity differed between the two sets of trials. Doing so reveals how the 3.33 mm difference in target position after that particular step influences participants’ ongoing movements. Determining how the difference in lateral *velocity* changes as a function of the time from the selected step is particularly useful for visualising the latency and vigour of the responses to such steps. We will therefore use the term ‘response’ to refer to this difference in velocity. For each moment of the step, we determined such responses for each participant, and plot the average of the 15 participants’ responses.

In addition to determining how the finger responds to steps at various moments we also determined several additional parameters. We defined the reaction time conservatively as the time it took for the finger to move 5 mm from its initial position after the target appeared (Brenner and Smeets 2019), and the movement time as the remaining time to the moment of the tap. Reaction time and movement time were determined for individual trials. For all such measures for which a single value was determined for each trial, we determined the median value for each participant and report averages of these median values with the associated standard deviations across participants ($\mu \pm \sigma$). In the figures we plot averages across participants with 95% confidence intervals.

We also report the average fraction of targets that participants hit with the associated standard deviations across participants. We determined the peaks of the responses for steps at various moments for each participant to examine whether changes in response vigour were similar across participants. Finally, we determined how steps at various times give rise to tapping errors by averaging lateral tapping errors (the signed difference between the position of the tap and the position of the target at the time of the tap) in relation to the direction of the step. Note that we use the term ‘error’ to refer to any deviation from tapping the target centre, irrespective of whether the target is hit. To select different moments we split the trials in the same way as we did for the analyses of the responses.

### Results

**Fig. 2:**
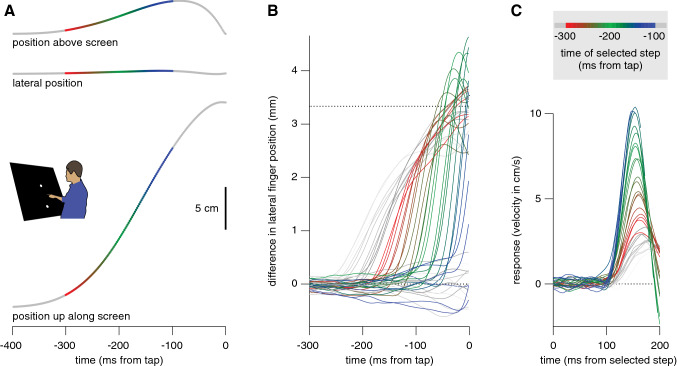
Results of Experiment 1, averaged across trials and then across participants. **A** Position of the finger along three dimensions as a function of the time before the tap. The colours provide a reference for the time of the selected step in the further analysis. **B** Difference between the average lateral position of the finger on trials with a rightward and leftward displacement of the selected step, as a function of the time before the tap. Separate curves show the influence of the direction of the step for different moments of the selected step (indicated by the curves’ colours). The dotted line at 3.33 mm indicates the position difference that matches the step size. Grey curves are shown with lower contrast as the time of the step changes from 300 to 400 ms before the tap (and 100 to 0 ms before the tap). **C** The response (time-derivative of the curves in **B**) for steps at different moments (indicated by the colours) as a function of the time from the selected step. The vigour of the response increases when there is less time left before the tap, while the latency does not change

Of the total of 7500 trials (15 participants, 500 trials each), we excluded 381 in which no tap was detected within 700 ms and 112 in which the finger’s position was invisible (primarily due to excessive supination of the arm) for longer than 20 ms during the movement. Participants hit 63 ± 9% of the targets. Given the conservative way of estimating reaction time, it was about what one would expect: 233 ± 21 ms. The movement time was quite short: 290 ± 58 ms. The average finger positions along each of the three dimensions during the last 400 ms before the tap are shown in Fig. 2A.

Irrespective of which step was used to separate the trials into two sets, separating the trials on the basis of the direction of a single step meant that the target positions differed by about 3.33 mm from that moment onwards (twice the step size; Fig. 1D). To hit the target, the finger positions should therefore differ by a similar amount by the time of the tap. The finger positions do differ by about 3.33 mm when there is enough time after the step (Fig. 2B).

To evaluate the latency and vigour of the response, we plot the response (which we defined as the difference in lateral velocity) as a function of the time from the selected step (Fig. 2C). We concentrated on responses for steps between 300 and 100 ms before the tap, because for earlier steps the finger had often not yet started moving by the time a response could be expected (9% of the movement times were shorter than 200 ms) and for later steps no response can be expected within the remaining time (Brenner and Smeets 1997). The latency of the response was a bit more than 100 ms, irrespective of the timing of the step (colours). The response was more vigorous when the selected step was closer to the time of the tap (gradient from red to blue). We confirmed that selecting steps with respect to when the finger started moving rather than with respect to the tap gave a similar pattern of responses (not shown).

**Fig. 3:**
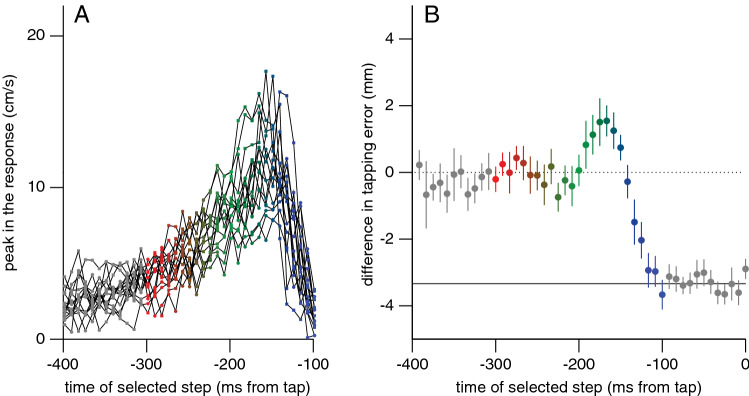
Additional results of Experiment 1. Colour coding as in Fig. 2. **A** Peaks in individual participants’ responses as a function of the time of the step. Lines connect points belonging to the same participant. Squares and circles represent participants with high and low overall peak responses, respectively (median split). **B** Systematic difference in tapping error between trials separated by the direction of a step, as a function of the time of the step. Steps that were more than 200 ms before the end of the movement are fully corrected for (dotted line). For steps between 200 and 150 ms before the tap the correction is too strong. Between 150 and 100 ms before the tap there is a partial correction. Steps less than 100 ms before the tap are not corrected for at all, so the error matches the step size (solid line). Error bars are 95% confidence intervals across participants (of the means of the individual mean values)

The observed increase in the vigour of the response when there was less time remaining until the tap was consistent across participants, as can be seen by plotting the peaks of individual participants’ responses as a function of the time of the selected step (Fig. 3A). Increasing the vigour of the response ensures that the difference in position caused by the step is covered in less time (Fig. 2B). The tapping errors show that this increase was effective for steps that took place more than 200 ms before the tap (Fig. 3B). Steps between 200 and 150 ms before the tap give rise to excessive corrections (larger adjustments than necessary: ending above the dotted line in Fig. 2B; positive values in Fig. 3B). The peak in the response is lower for later target steps (Fig. 3A), because for such steps the tap occurs while the response is still increasing (Fig. 2C). Consequently, target steps that occur later than 150 ms before the tap cannot fully be accounted for (negative values in Fig. 3B). Since there is a latency of about 100 ms to respond to a step (Fig. 2C), there is no response to steps that occur during the last 100 ms (grey curves that do not rise in Fig. 2B). Consequently, selecting steps during the last 100 ms reveals a negative difference in tapping error of twice the step-size (Fig. 3B).

### Discussion

The responses shown in Fig. 2C are similar to responses found in experiments that use a single target step (Brenner and Smeets 1997; Zhang et al. 2018). Similar responses are also found for the leg rather than the arm (Zhang et al. 2000). Studies with isolated target steps had already shown that the response is more vigorous if there is less time available after the step (Gritsenko et al. 2009; Liu and Todorov 2007; Oostwoud Wijdenes et al. 2011; Zhang et al. 2018). It has also been shown that the response to a step is not influenced by a step having occurred 200 ms earlier (Oostwoud Wijdenes et al. 2011). We show that both these characteristics also apply when many target steps occur during a single movement, indicating that they describe how humans deal with noisy position information.

Despite the additional uncertainty about the target’s position as a result of random jitter, we see no indication of position information being accumulated over extended periods of time. Given that one cannot even start to adjust one’s movements to deal with steps that occur 100 ms before the tap (rightmost blue dot in Fig. 3B) and that one can fully correct for steps that occur only 50 ms earlier, any accumulation of information would have to be fast enough to completely replace the estimated position well within 50 ms. It is very unlikely that sensory judgments contribute much to the 50 ms that is needed to adjust to the steps, because there are also obvious mechanical constraints on how quickly the required adjustments can be made (as for instance captured by the mass-spring-damper model that we will discuss below).

One might argue that the drift in target position as a result of the target following a random walk makes it disadvantageous to accumulate information about target position, because at any moment the latest value is the best predictor of future positions. However, we believe that this is actually quite representative of motor control in daily life, because the egocentric positions of static objects are normally always varying a bit due to postural sway and other small movements of the observer (Aytekin and Rucci 2012). Such variations in egocentric position are more similar to a random walk than to random fluctuations around an average. Moreover, we know that people do not readily take the likelihood of targets changing position into account. For instance, the response to a target jump remained unaltered after multiple trials in which it always jumped back after 150 ms (Brenner et al. 2022).

**Fig. 4:**
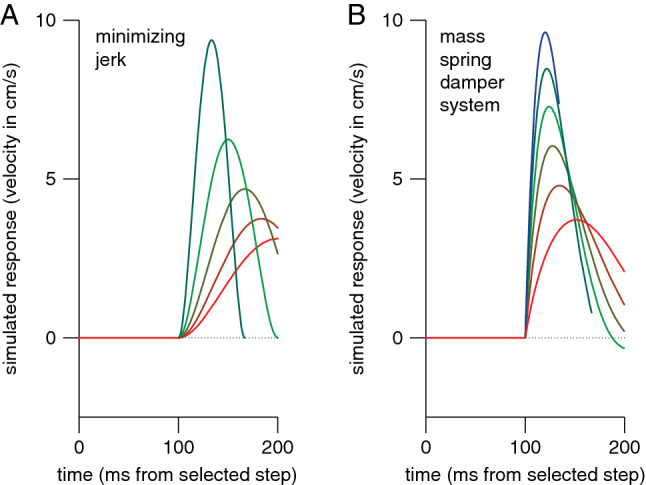
Two simple models of the responses to a target displacement (D) of 3.33 mm (as shown in Fig. 2C). For both models, the time available for the response (T, in ms) starts 100 ms after the displacement. **A** Minimal jerk trajectories that would precisely traverse D in time T, for five moments of the selected step (colour coded as in Fig. 2). The equation is $\dot{x}=\frac{30D{t}^{2}{\left(1-t\right)}^{2}}{T}$, where t is time as a fraction of T. Trying to adjust for the step as smoothly as possible within the remaining time leads to an increase in vigour as the time until the tap (T) decreases. **B** Responses of a mass-spring-damper system that stiffens linearly during the movement. We used $\ddot{x}+b\dot{x}+kx=0$ to predict the response to a displacement D, with ${b=75 s}^{-1}$ and $k=3800-16T {s}^{-2}$. This system is underdamped for T < 150 ms (green and blue curves), leading to an overshoot and negative velocities later in the response unless the movement ends before that

To further evaluate the credibility of responses being guided by instantaneous information about the target’s position, we considered whether the responses might be close to optimal in any particular respect. Since we only measured the position of the tip of the finger, and participants could stand and move as they pleased, we cannot determine any mechanical or physiological measure of optimality. We can, however, predict what the kinematics of the tip of the finger would look like if the smoothness of the adjustments were optimised (minimum jerk model; Flash and Henis 1991; Flash and Hogan 1985; Wong et al. 2021). When there is more than 200 ms available between the selected step and the tap, the observed adjustments resemble what one would find if one were to adjust the movements as smoothly as possible (Fig. 4A). When there is less time left, the actual corrections can no longer be completed in time (Fig. 3B). The minimal jerk model does not reproduce this because it has no mechanical limitations. However, finding that many movements are adjusted as smoothly as possible supports the idea that other constraints than the accumulation of information about the target’s position determine the time constant of the response.

A somewhat realistic way to consider mechanical constraints when modelling adjustments to movements towards a jittering target (in a simple manner; Smeets and Brenner 2002) is by using a mass-spring-damper model (de Lussanet et al. 2002; McIntyre and Bizzi 1993; Smeets and Brenner 1995a). This approach is based on the idea that any biological non-linear system can be approximated by a linear system within a small range. Modelling the adjustments as the finger being pulled by each small target step with a constant damping and a stiffness that increases during the movement reproduces several critical aspects of the response (Fig. 4B). The increasing stiffness gives rise to the observed increase in vigour for responses to later steps, with a higher peak velocity being reached at a slightly earlier moment. The increasing stiffness also means that responses to later steps are under-damped, and therefore overshoot the target if there is enough time, resulting in errors that are systematically in the opposite direction than the step for steps between 200 and 150 ms before the tap, as can be observed in the data (Fig. 3B).

The model predictions in Fig. 4 use instantaneous visual information in combination with either kinematic or mechanical constraints on the way participants respond. The fact that these models reproduce critical characteristics of our participants’ responses supports the idea that constraints on how participants respond, rather than an accumulation of visual information, underlie the smooth nature of the response. We therefore conclude that our participants continuously use the latest position information.

### Experiment 2: velocity

In Experiment 1 we found no evidence that position information was accumulated over extended periods of time. Participants appeared to adjust their movement to the latest available information about the target’s position. For a target that follows a random walk (as was the case in Experiment 1), the instantaneous position is indeed the best estimate of its future position. Considering past positions, for instance by considering the direction of the latest displacement, is of no help in determining where one can best try to hit the target.

When trying to hit a target that is consistently moving in a certain direction, one anticipates where it can be hit by extrapolating from its current position with its estimated velocity until the estimated moment of the hit (Brenner and Smeets 2018; de la Malla et al. 2018). In order to estimate velocity, the visual system needs to combine information across tens of milliseconds (van Doorn and Koenderink 1982). Precision improves as more information is provided for up to 100 ms (Snowden and Braddick 1991). If the target’s velocity is constant, accumulating information for a longer time period could potentially provide a more precise estimate of the target’s average velocity, and therefore a more precise extrapolation. But if the velocity is not constant, longer accumulation will introduce biases. Is the target velocity that one uses for the extrapolation accumulated across more time than is strictly necessary? Is the position at which one anticipates to hit the moving target accumulated, in accordance with the use of accumulated velocity information to estimate that position, or does the extrapolated position (towards which the hand is guided) still follow the instantaneous position of the target?

To find out, we added a 5 mm rightward displacement to each step of the random walk that was used in Experiment 1. This corresponds with adding a rightward velocity of 60 cm/s to the jitter. Adding rightward motion to the jittering target makes the jitter hardly noticeable, which might make it less evident that relying on the latest position is beneficial. In Experiment 2 we examined whether the responses to steps in target position are different when the target moves systematically to the right, as well as how information about the target’s velocity is accumulated to predict the position at which it will be intercepted.

### Methods

Twenty adults took part in Experiment 2. None of them had taken part in Experiment 1. The experiment was identical to Experiment 1, except that the target appeared 20 cm further to the left and was displaced by an additional 5 mm to the right on each frame so that on average it moved rightwards at 60 cm/s. Thus, the 1.67 mm leftward or rightward target steps of Experiment 1 were replaced by small (3.33 mm) or large (6.67 mm) rightward steps, corresponding with velocities of 40 or 80 cm/s. There was no time limit for hitting the target other than that it had to be hit before moving off the screen. To determine the tapping error, the position of the target at the moment of the tap was assessed using linear extrapolation during the step in question. This was done during the experiment, because the error was also used to provide feedback: the target stopped at its position at the time of the tap if it was hit, and moved away from the finger from that position if it was missed.

We analysed the influence of the change in position as a result of each step in the same way as in Experiment 1, and compared the results to those of Experiment 1 to look for evidence of more accumulation of information. The response was now the difference between average lateral finger velocities after splitting the data by the size rather than the direction of the step. To evaluate whether the estimate of velocity is accumulated across more than tens of milliseconds, we examined to what extent steps early during the trial influence the extrapolation later during the trial, and thereby the tapping error.

The target’s position and velocity during a trial are not independent, making it difficult to separate influences of estimates of the two attributes. The target’s position and velocity are, however, independent of those in the previous trial. To explore whether information about the target’s velocity is accumulated across hundreds of milliseconds, we also examined influences across trials. Randomly choosing step sizes from when the target appeared until the tap meant that the target’s mean velocity differed across trials (with a standard deviation of 2.6 cm/s). We examined whether the mean velocity on the previous trial influenced where participants tapped. To do so, we split the data on the basis of whether the mean velocity on the previous trial was higher or lower than 60 cm/s (ignoring trials if the previous mean velocity was exactly 60 cm/s). Assuming that the influence of the target’s velocity on the previous trial is the result of information about the target’s velocity accumulated over time, we expect to see a clearer influence of the previous target’s velocity when we split the data by the mean velocity during the last 200 ms of the previous trial. However, the target’s velocity during the last part of the movement inevitably influences the tapping errors (Fig. 3B). To evaluate to what extent a clearer influence of the velocity at the end of the previous trial is the result of accumulating velocity information rather than of responding to errors associated with that velocity, we also split the data on the basis of whether the tap was to the left or to the right of the target centre on the previous trial. In all these cases, we compared tapping errors on the current trial, after splitting the trials on the basis of the velocity or error on the previous trial.

### Results

**Fig. 5:**
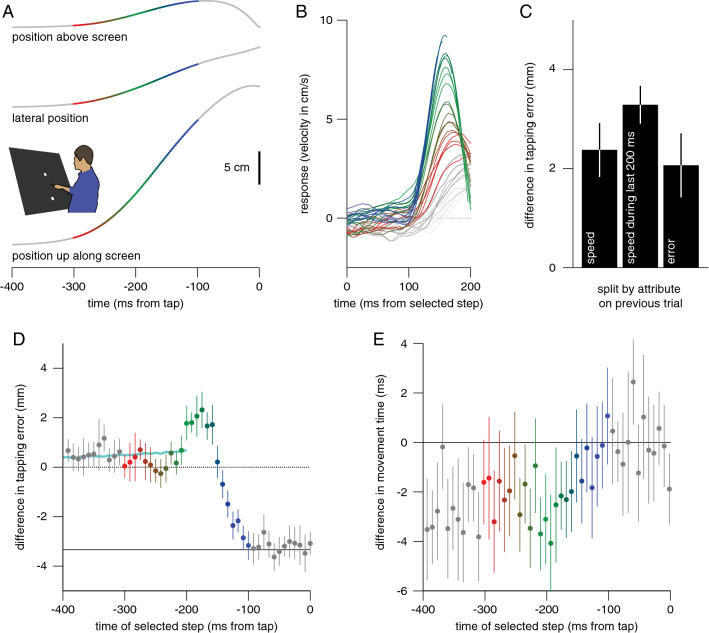
Results of Experiment 2. **A** Position of the finger along three dimensions as a function of the time before the tap. **B** Velocity of the lateral response to steps at different moments as a function of the time from the selected step. **C** Systematic difference in tapping error between trials in which the target moved faster or slower than average during the previous trial, moved faster or slower than average during the last 200 ms of the previous trial, and in which the finger ended to the right or left of the previous target. **D** Systematic difference in tapping error as a function of the time of the step. The turquoise curve shows the pattern of differences in error that one might anticipate on the basis of the model proposed in Eqs. (2) and (3). **E** The difference between the mean movement times on trials with a large and a small step as a function of the time of the selected step. Error bars are 95% confidence intervals across participants

Of the total of 10,000 trials (20 participants, 500 trials each), there were 15 in which no tap was detected and 46 in which the marker was invisible for longer than 20 ms during the movement. All other trials could be used for the analysis. Both the reaction time (209 ± 17 ms) and the movement time (280 ± 36 ms) were slightly shorter than in Experiment 1 (as expected for moving targets; Smeets and Brenner 1995a). The fingers’ trajectories (Fig. 5A) were similar to those of Experiment 1 (Fig. 2A), except for moving to the right in accordance with the fact that the target was about 10 cm to the right of the screen centre at the time of the tap.

The pattern of responses to the selected steps (Fig. 5B) is very similar to that in Experiment 1 (Fig. 2C), except that the initial 100 ms of the response is not centred on zero (with some random variability). Especially for steps that took place more than 200 ms before the tap (red curves), the response is initially slightly negative and increases gradually. This obviously cannot be a response to the step because there is no latency, so it must arise from the way we synchronized and selected the movements. Indeed, if the trials are aligned with respect to the reaction time rather than with respect to the time of the tap no such differences are observed for responses to steps at similar times during the movement (not shown). We return to this issue in the discussion. Importantly, we found no evidence that accumulation of position information has made the responses to target steps less vigorous for a moving target: participants adjusted their movement in accordance with the instantaneous position in the same manner as for the targets that did not move consistently (Experiment 1).

As one would expect if information about the target’s velocity is accumulated across hundreds of milliseconds, step sizes early in the trial influenced the tapping error (leftmost 17 symbols above zero in Fig. 5D). When the step size was large, participants tapped further ahead of the target, which is consistent with using a higher estimate of target velocity for extrapolating to the time of the hit. We will analyse these errors more quantitatively in the discussion section. To check whether the velocity during the previous trial also influenced the estimate of velocity that was used to extrapolate the target’s motion, we split the data on the basis of the average speed of the target during the previous trial. This split resulted in a difference between the tapping errors on the current trial of about 2.4 mm (leftmost bar in Fig. 5C). In accordance with the estimate of target velocity resulting from accumulating information over time, splitting the trials on the basis of the previous target’s average speed during the last 200 ms of its motion resulted in a larger difference between the errors: about 3.3 mm (second bar in Fig. 5C). Is the difference in errors really due to accumulation of velocity across trials, or can it be explained by participants aiming further ahead of the target on trials preceded by ones that end with many large steps because such steps make one hit behind the target (rightmost grey symbols in Fig. 5D)? To find out, we also split the data on the basis of the tapping error in the previous trial. We found that participants tapped about 2.1 mm further to the right after having tapped to the left of the target centre than after having tapped to the right of the target centre on the previous trial (rightmost bar in Fig. 5C). This difference is smaller than the 3.3 mm effect of the final velocity, so correcting for the error on the previous trial is probably not the only reason that the velocity on the previous trial has an effect. We will return to the effect of errors in the previous trial in Experiment 5.

In our analysis, we concentrate on the lateral component of the finger’s movement. We observe that when the target made a large rightward step, the finger was adjusted to the right. To compensate for the target moving faster when the step is large, participants could also move their finger faster. Indeed, participants not only adjusted where they tapped, but also when they tapped: until about 150 ms before the tap, a large step made participants tap about 3 ms earlier than a small step (Fig. 5E). This could be because people adjust the speed of their movement when the velocity of the target changes (Brenner et al. 1998), but adjusting the movement speed took about 200 ms in that study, whereas the step size continued to influence the movement time until about 100 ms before the tap in the current experiment (Fig. 5E), so the mechanism might be different.

### Discussion

The way in which the finger responded to the selected step (Fig. 5B) is quite similar to the way it did in Experiment 1 (Fig. 2C). The way in which tapping errors depended on the time of the step (Fig. 5D) was also quite similar to the way they did in Experiment 1 (Fig. 3B). This implies that people rely on the target’s instantaneous position despite the added lateral motion. There are two trends in the data of Experiment 2 that were not observed in Experiment 1, and that therefore need to be discussed: the small but evident response during the first 100 ms after early steps (the gradual shift upwards of the red curves in Fig. 5B) and a tendency to tap further ahead of the target after large steps early in the movement in Fig. 5D (first 17 points all above zero).

The apparent response during the first 100 ms after the step in Fig. 5B obviously cannot be an actual response because its latency is too short. It is presumably an artefact of the movement time also being adjusted to the step size to some extent (Fig. 5E). A consequence of adjusting the movement time is that when comparing steps at the same time before the tap (which is what we do in Fig. 5) one is comparing steps longer after movement onset for the small step than for the large step. Since the finger is moving to the right (Fig. 5A) this timing difference results in the finger initially being less far to the right for the trials with large steps.

#### Modelling velocity accumulation

As already mentioned, the tendency to tap further ahead of the target after large steps early in the movement (Fig. 5D) probably arises from information about the target’s velocity being accumulated across hundreds of milliseconds. We used a simple model of where participants are likely to tap when faced with a visuomotor delay to quantify this idea (based on Eq. 1 of de la Malla et al. 2018). The delay of 100 ms that was used in that paper is consistent with what one would estimate from Figs. 2C and 5B); we will refer to this delay as δ. The idea of this model is as follows:

At time *t* participants will be aiming at a position (${A}_{t}$) that is determined by extrapolating from the estimated target position a visuomotor delay earlier (${T}_{t-\updelta }$) with an estimate of the target’s velocity at that time (${v}_{t-\updelta }$) for the duration between the time when this estimate was obtained ($t-\updelta )$ and the anticipated time of the tap (${t}_{tap}$):

1 $${A}_{t}={T}_{t-\updelta }+{v}_{t-\updelta }\left({t}_{tap}-\left(t-\updelta \right)\right).$$

We can use this position to predict the tapping error, assuming that participants know the time of their tap and reach the position they are aiming for. It is the difference between the position that participants are aiming for and the target position at the time of the tap:

2 $${A}_{tap}-{T}_{tap}={T}_{tap-\updelta }-{T}_{tap}+{v}_{tap-\updelta }\cdot\updelta .$$

We next introduce a very simple model for how participants could determine *v* by accumulating information about the target’s velocity across time. The results shown in Fig. 2C and D show that the accumulation extends over hundreds of milliseconds, including the previous trial. We propose that our participants update the estimated velocity (*v*) after each new step (*s*) with a weight of 2% being given to the velocity of that step (${v}_{step}$), so that the estimated velocity on step *s* is

3 $${v}_{s}=0.98 {v}_{s-1}+0.02 {v}_{step}.$$

The weight given to the velocity on the latest step (0.02) determines the rate at which the estimate of velocity is updated. Since there were 120 steps per second, the visuomotor delay $\updelta$ of 100 ms corresponds with 12 steps. We can combine Eqs. 2 and 3 to examine to what extent the proposed accumulation of velocity information could explain the observed systematic errors (Fig. 5D). We ran 1000 simulations of sessions of 500 different trials. On each simulated trial the initial value of *v* was the value at the end of the previous trial, and this value was updated by the number of steps that were shown in a randomly selected trial of the experiment (so the mean and variability in the number of steps was the same for the experiment and for the simulations). The results are shown as a turquoise curve in Fig. 5D. The approximate match with the data implies that slowly updating the estimate of velocity could account for the observed tendency to tap further ahead of the target after large steps early in the movement. Since it only makes sense to use an estimate of target velocity to predict a future target position if the target is moving systematically in a certain direction, this interpretation is consistent with there being no tendency to tap further ahead of the target after large steps early in the movement for the randomly jittering targets of Experiment 1 (Fig. 3B).

In the model, each new step contributes 2% to the velocity estimate (Eq. 3). When updating the estimate at this rate it takes almost 300 ms to reach halfway to a new value after an abrupt change. Such slow updating is needed to explain why the velocity on the previous trial influences one’s movements (Fig. 5D) under the assumption that the initial estimate on each trial is the final value of the previous trial. Analysing the influence of the velocity on the previous trial (Fig. 5C) in a similar manner for the simulated sessions as for the actual data predicts an influence of 1.2 mm for the present Experiment. This value is only half of the 2.4 mm that we found, but the discrepancy does not necessarily mean that the model (or the updating rate of 2%) is wrong, because in the actual experiment correcting for the error on the previous trial might also contribute to the influence of the previous trial. We will add error-based corrections to the model when modelling the results of Experiment 5, in which the targets’ velocities changed systematically.

## Experiment 3: initial velocity

In Experiment 2 the jitter in the target’s position was no longer conspicuous because of the overall rightward target motion. Moreover, the target’s velocity had to be considered to anticipate where the target could be hit. Nevertheless, the adjustments to the steps were very similar to those in Experiment 1. The main difference was that the tapping errors depended to some extent on steps more than 200 ms before the tap in Experiment 2. We attributed this effect of early steps to accumulating velocity information to predict the moving target’s position at the time of the tap. However, since the targets always started moving at the same position, a higher velocity meant that the target was also further to the right at that time. To confirm that it is the velocity of the target that influences the tapping error, rather than an associated influence on the position, we performed a modified version of Experiment 2 in which we did not rely exclusively on random variations in velocity, but let each trial start with 100 ms of either fast or slow target motion. The targets’ starting positions were adjusted so that they reached the same position on the screen after the initial 100 ms. If the velocity of the target is constantly accumulated as proposed in Eq. 3, this initial period of faster or slower movement should influence the tapping errors. If only the lateral position at the time of the tap is relevant it should not.

### Methods

Eight adults took part in the experiment, several of whom had taken part in Experiment 1. None had taken part in Experiment 2. The experiment itself was identical to Experiment 2 except that the first 12 steps of the target’s motion on each trial were either all small (3.33 mm) or all large (6.67 mm), leading to 100 ms of target motion that was either slow or fast (250 trials each). Targets that initially moved slowly (40 cm/s) appeared 18 cm to the left of the centre of the screen. Targets that initially moved fast (80 cm/s) appeared 22 cm to the left of the centre of the screen. After the first 100 ms, all targets were therefore 14 cm to the left of the centre of the screen. From then on, the step size was selected at random as in Experiment 2. Consequently, any systematic difference between the errors for the two initial velocities must be due to the motion during the initial 100 ms. We examined how the velocity of the first 12 steps influenced tapping errors. On the basis of our model (and tapping almost 500 ms after targets appeared) we expected to find a difference of several mm between trials in which the steps were initially large and small. We also examined the responses to target steps at different moments and systematic tapping errors for steps at different moments as in the previous experiments. Systematic tapping errors for steps at different moments were also compared with predictions based on the proposed model of velocity accumulation, as in Experiment 2.

### Results and discussion

**Fig. 6:**
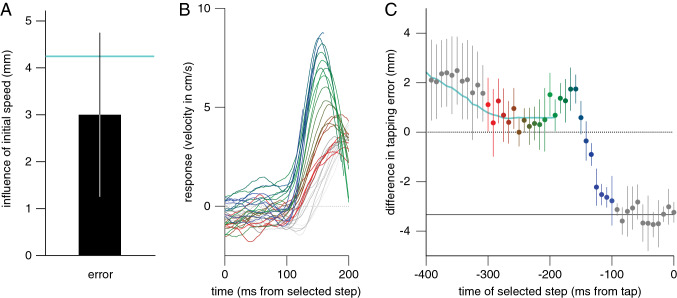
Results of Experiment 3. **A** Systematic difference between the tapping errors when there were only large or only small steps during the first 100 ms of target motion. **B** The response to a target step as a function of the time after the step. **C** Systematic error in the tap as a function of the time of the step. The turquoise line and curve show the anticipated difference in error for simulations based on Eqs. 2 and 3. Error bars are 95% confidence intervals across participants

Of the total of 4000 trials (8 participants, 500 trials each), there were 8 in which no tap was detected and 177 in which the finger’s position was invisible for longer than 20 ms during the movement. On the remaining trials, the reaction time was 211 ± 14 ms when the target’s initial velocity was slow, and 210 ± 13 ms when it was fast. The movement time was 283 ± 46 ms when the target’s initial velocity was slow, and 282 ± 49 ms when it was fast. At the end of the initial 100 ms, the targets’ positions were matched. Subsequent step sizes were chosen at random. We can therefore directly evaluate how the initial target velocity influenced where participants tapped by comparing errors when the target moved slowly during the first 100 ms, to those when the target moved fast. Participants tapped about 3 mm further to the right (with respect to the target) when the target moved faster during the first 100 ms (Fig. 6A).

Not surprisingly, the responses to the random steps were very similar to the responses in Experiment 2 (compare Fig. 6B with Fig. 5B), including the negative bias during the initial 100 ms. The influence of step size on the error during the last 300 ms before the tap was also very similar to that in Experiment 2 (compare Fig. 6C with Fig. 5D). For selected steps that were more than 300 ms before the tap, the errors were clearly larger here than in Experiment 2. This is because the steps during the first 100 ms of each trial are not independent. The pattern of errors that arises from the correlation between early steps is captured quite well by the model simulations that rely on slowly accumulating velocity information (Eqs. 2 and 3; turquoise curve in Fig. 6C). Since we did not change anything in the simulations other than the stimuli (the first 12 steps always having the same size), the fact that the simulations are so consistent with the actual differences in tapping errors for steps between 400 and 200 ms before the tap in Experiment 3 (Fig. 6C) as well as Experiment 2 (Fig. 5D) supports the idea underlying the model: that the velocity that is used to extrapolate to the position of the hit is accumulated over time.

## Experiment 4: variability in step size

In our interpretation and modelling of the data of Experiments 1–3 we implicitly assumed that the response to a step is proportional to the step size, and that the accumulation of information is independent of the step size. To evaluate whether these assumptions are justified we compared responses to steps of different sizes, and examined how the variability in step size (the amplitude of the jitter) affects the tapping errors that arise from having an initial 100 ms of fast or slow target motion. If participants adjust the extent to which they accumulate velocity information to the variability in velocity, we expect them to consider less time when the velocity is less variable, so we expect to see a smaller effect of the first 100 ms when there is less variability in step size. We therefore repeated Experiment 3 using blocks of trials in which the step sizes differed to different extents after the first 100 ms.

### Methods

Twenty-four adults took part in the experiment, none of whom had taken part in any of the other Experiments. Each participant took part in four blocks of 100 trials, with a short break between the blocks. Within each block, half the targets initially moved for 100 ms at 40 cm/s (slow) and the other half initially moved for 100 ms at 80 cm/s (fast). After that, the target velocity was chosen at random from two values for each step, as in Experiments 1–3. These two values differed between blocks. The difference between the two step sizes was either absent (*none*; the target simply moved at 60 cm/s), smaller than the difference in the other experiments (*small*; steps corresponding with moving at 50 or 70 cm/s), the same as in the other experiments (*standard*; 40 or 80 cm/s), or larger than in the other experiments (*large*; 25 or 95 cm/s). The four blocks were presented in a counterbalanced order such that each possible order was used once. Further experimental details were identical to those of Experiments 1–3, except that the target was slightly larger (radius of 1.2 cm rather than 1 cm).

Two new analyses were added to the analyses that we had conducted for Experiment 3. The first is that we used a repeated measures analysis of variance to help evaluate whether the variability in step size (none, small, standard or large) affected how much the target’s velocity during the first 100 ms influenced the tapping errors. The second is that we compared the peak responses across the three conditions with different step sizes in much the same way as we did the peak responses of individual participants in Experiment 1 (Fig. 3A). For the latter analysis, we defined the gain of the response as the response divided by the step size. We averaged the gain across participants before determining the peak. If the response is proportional to the step size, the gain should be similar in the different blocks (for any time of the step).

### Results and discussion

We planned a total of 9600 trials. We lost 40 trials because the first two participants accidentally received too few trials in the block with the standard variability (40 rather than 50 for each initial velocity). In 323 trials no tap was detected online. Of these, 301 could be recovered because the participant simply tapped too gently (we considered the minimum in the height of the finger with respect to the screen to be the moment of the tap). The remaining 22 trials had to be excluded because there did not appear to be any tap (the finger was never close to the screen near the target’s path). We also excluded 94 trials that had questionable timing (there was reason to believe that the synchronization had failed because the light sensor registered an additional flash).

**Fig. 7:**
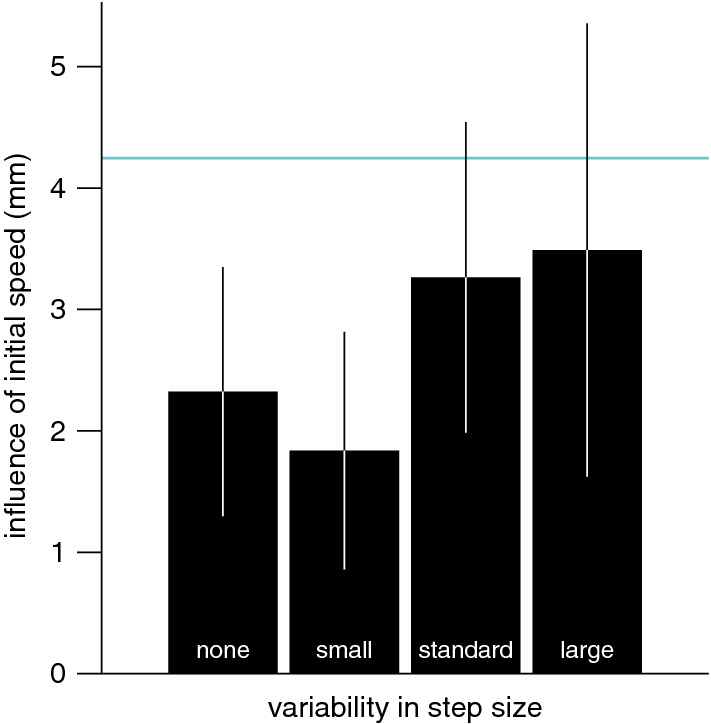
Difference between tapping errors when the target moved at 40 or 80 cm/s during the first 100 ms in Experiment 4. The influence is shown separately for the four blocks that differ in the step sizes used after the initial 100 ms. The turquoise line indicates the same model prediction as in Fig. 6A. There was a clear influence of the initial speed in all blocks. The tendency for the influence to be smaller in blocks with less variability between the step sizes was not statistically significant. Error bars are 95% confidence intervals across participants

The reaction time was 210 ± 20 ms for the block in which the target moved with no variability, 207 ± 18 ms for the block with low variability, 213 ± 24 ms for the block with standard variability, and 211 ± 24 ms for the block with high variability. The movement times were 267 ± 42 ms, 272 ± 43 ms, 272 ± 45 ms, and 262 ± 52 ms, respectively. Participants tapped further ahead of targets that moved faster during the first 100 ms (positive values in Fig. 7). In accordance with participants considering the velocity during less time (updating it faster) when the velocity is less variable, we see a smaller effect of the first 100 ms when there is less variability in step size, but the difference in effect size between the blocks is not statistically significant (F_3,69_ = 1.43, *p* = 0.24). For the equivalent block (standard variability), the influence of the initial speed (3.3 mm) was similar to that in Experiment 3 (3.0 mm).

**Fig. 8:**
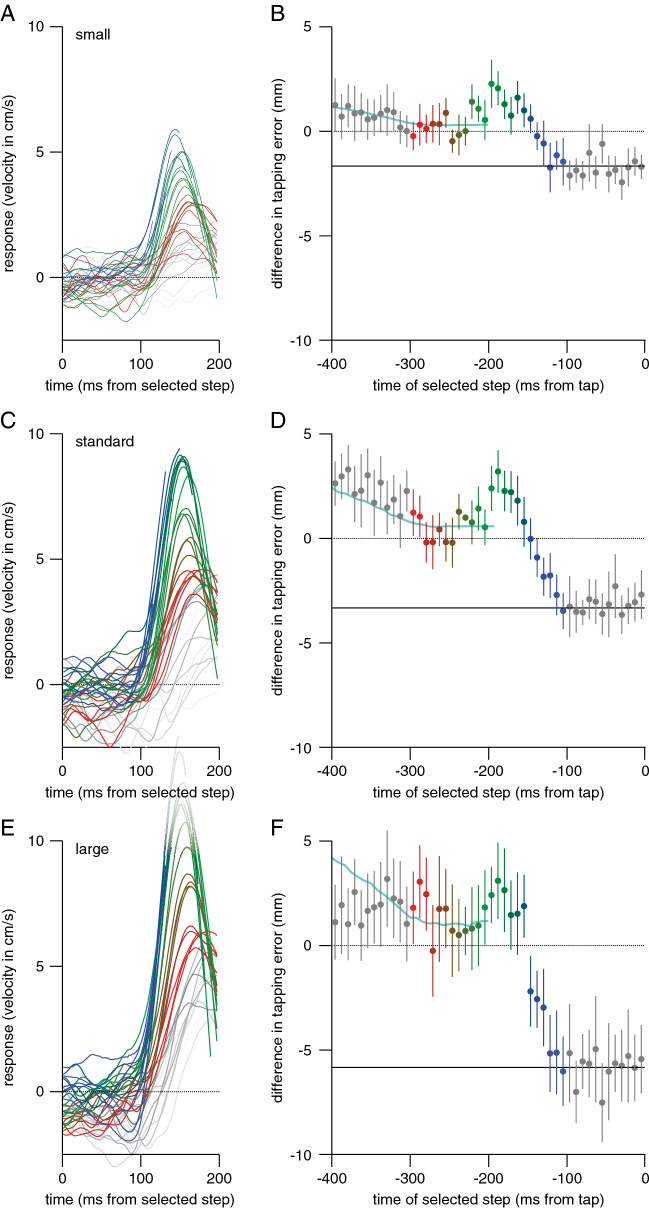
Additional results for the blocks with small, standard and large variability in step size in Experiment 4. **A**,** C**,** E**. Responses to target steps as a function of the time after the step. **B**,** D**,** F**. Systematic tapping errors as a function of the time of the step. The turquoise curves show similar model predictions to those in Figs. 5D and 6C. **A**,** B**. Small variability. **C**,** D**. Standard variability. **E**,** F**. Large variability. Error bars are 95% confidence intervals across participants

**Fig. 9:**
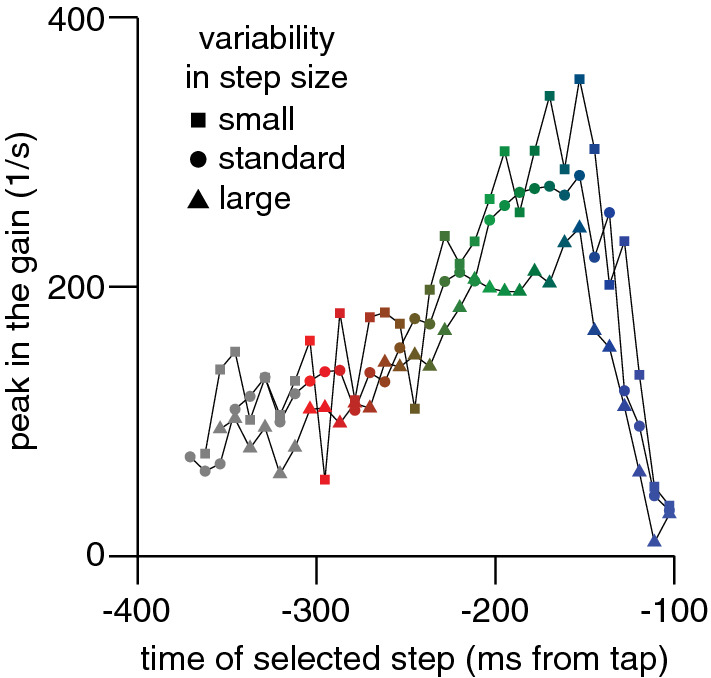
Vigour of responses to steps of various sizes (different symbols) as a function of the time of the step in Experiment 4. The peak in the gain is defined as the peak in the response (Fig. 8A, C and E) divided by the step size

The responses to the selected steps were similar to those of Experiments 1–3, except that their magnitudes scaled with the differences between the step sizes. When the variability in step size was smaller (Fig. 8A) or larger (Fig. 8E), the responses were too. The scaling with step size is shown more clearly in Fig. 9, which shows the values of the peaks in the responses to steps at various times as a function of the time of the step (as in Fig. 3A, but averaged across participants), after dividing the peaks by the step size to obtain a ‘gain’. The peaks in the gain are very similar for the three amounts of variability, except that the values for the high variability are lower than the others when the peak in the gain is high (green symbols). This probably reflects a mechanical constraint on how vigorously the hand can respond to the perturbation. The decline in the peak gain between − 150 and − 100 ms from the tap (blue symbols) is caused by the movement terminating before the response can reach its peak (see Fig. 8).

The steps at various moments influenced the errors in accordance with the model predictions: errors were smaller for smaller steps and larger for larger steps (Fig. 8B, D, F). The only clear discrepancy between the data and the model is that the influence on the errors appears to be smaller than predicted for large steps long before the tap (leftmost points in Fig. 8F). A smaller influence of early steps (but not slightly later steps) is indicative of a shorter duration of accumulation (faster updating of velocity information), so finding this for the block with the highest variability contradicts the idea that the slow updating of velocity information is a response to the high variability in target speed. The fact that the results for the block that replicates Experiment 3 (*standard*) match the data of Experiment 3 very well (compare Fig. 6B, C with Fig. 8C, D), despite the fact that these were completely different participants, makes us confident that the overall pattern is reproducible.

## Experiment 5: acceleration

We found in Experiments 2–4 that information about a target’s velocity was accumulated across hundreds of milliseconds. Doing so only makes sense if the velocity does not change systematically during that time. This was the case in Experiments 2–4, but it is not generally the case in daily life. For example, consider catching a falling ball. Gradually accumulating information about the falling ball’s velocity will make one systematically underestimate its velocity near the moment of the catch. People can catch falling balls, so in our final experiment we tried to determine what people learn when they learn to deal with acceleration.

One option would be to learn not to accumulate velocity information as gradually when the velocity is changing systematically. Adjusting how velocity information is accumulated to whether and how the target’s velocity is changing requires the ability to quickly judge the extent of such changes. People are very poor at visually judging acceleration (Brouwer et al. 2002; Calderone and Kaiser 1989; Gottsdanker et al. 1961; Watamaniuk and Heinen 2003; Werkhoven et al. 1992) and do not consider visual information about acceleration to guide their actions (Benguigui and Bennett 2010; Brenner and Smeets 2015; Lee et al. 1997, 1983; Port et al. 1997). Rather than relying on visually judged acceleration to determine how gradually to accumulate visual information about the target’s velocity, people might consider how likely it is that the velocity is changing under the prevailing circumstances to do so. For example, they might consider it unlikely that a falling ball has a constant vertical velocity, because they have ample experience with gravity (Jorges and Lopez-Moliner 2017). They probably do not even need so much experience, because knowing the circumstances is not always as useful as repeatedly experiencing those circumstances (Orban de Xivry and Lefevre 2016). If targets accelerate in the same way across a few trials, participants learn to compensate for the systematic errors that arise from ignoring the acceleration (Brenner et al. 2016). They presumably do so by adjusting their actions in a manner that reduces errors on subsequent trials, rather than by reducing the time across which they accumulate information about the target’s velocity, because when the acceleration changes they have to readjust to the new acceleration.

In our final experiment, we examined how regularities across trials help people cope with consistent changes in target velocity. We presented participants with various target trajectories: accelerating or decelerating, moving at one of two different initial velocities, either to the left or to the right. When presented with a sequence of accelerating targets, participants can learn to compensate for the systematic error that arises from ignoring the increase in velocity by aiming slightly further ahead of the target than the position they estimated on the basis of the judged target position and velocity. Importantly, if participants simply learn to compensate for errors, then when presented with a mixture of accelerating targets that are moving to the right and decelerating targets that are moving to the left they should be able to compensate for systematic errors that arise from ignoring the changes in velocity by tapping further to the right of the target than estimated on the basis of the judged target position and velocity. Can participants do so?

### Methods

Seventeen adults including two of the authors each took part in two sessions separated by a short break. Several of these participants also took part in Experiments 1 and 3. The equipment, task, stimuli and procedure were the same as in Experiments 1–4, unless mentioned otherwise. An important difference was that the target velocity was constantly increasing or decreasing on each trial, rather than fluctuating around a fixed value. The target’s radius was 1.5 cm. The starting point was 30 cm below the target’s path, rather than 25 cm.

**Fig. 10:**
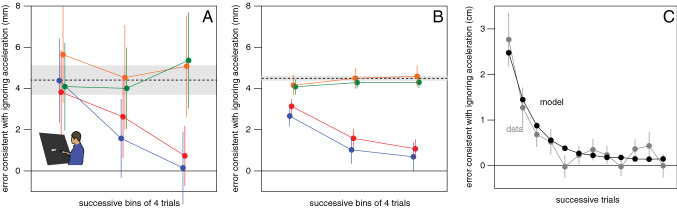
Extent to which participants were able to limit systematic acceleration-related errors in various blocks of 12 trials. **A** Results of Experiment 5. Dashed line: average systematic error across all reference blocks. Symbols: average systematic error across bins of four consecutive trials for each pair of blocks (colour coded as in Table 1). Values are means with 95% confidence intervals (grey area and error bars) across participants. **B** Results of a simulation of how people might learn to compensate for errors in Experiment 5 (see Eq. 5). Points and dashed line show medians and range containing 95% of the values for 1000 simulations of the whole experiment. **C** Experimental data (grey symbols) of a similar experiment in which targets moved to the right and either accelerated or decelerated in alternating blocks of 12 trials (data from Brenner et al. 2016). The errors are consistent with simulations based on the same model as was used to obtain the values in B (black symbols). In this panel the dots represent successive trials rather than bins of 4 consecutive trials

We presented participants with eight possible target trajectories (Table 1). Targets could be moving to the left or to the right, could either accelerate or decelerate at 40 cm/s^2^, and could move relatively slowly or relatively fast (differences in velocity of about 14 cm/s). Slower targets appeared 30 cm from the screen centre and faster targets appeared 40 cm from the screen centre, always initially moving towards the screen centre. Their initial velocities were chosen such that they would always be 32.5 cm from the edge of the screen after 700 ms. Since we expected participants to tap in less than 700 ms, this ensured that all targets could be hit, irrespective of their trajectory. However, we did not instruct participants to tap within 700 ms (Fig. 10).

**Table 1:**
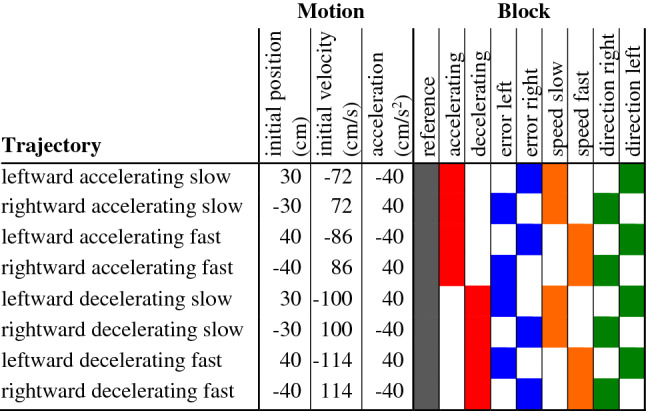
Details of the target’s motion for each of the eight kinds of trajectory, and schematic of the combination of trajectories in each of the nine kinds of blocks

Except for the reference block, that contained all eight kinds of trajectories, each block contained four kinds of trajectories, colour coded for quick reference to Fig. 10. The reported positions and velocities are the initial values. All values only refer to the lateral component, with positive values being to the right (with respect to the screen centre for the initial position)

A session consisted of 168 trials, presented in 17 blocks. Eight of these blocks constituted the heart of each session (eight rightmost columns in Table 1). These experimental blocks can be regarded as four pairs (see colour coding in Table 1). In a block of each pair, four of the eight possible trajectories were each presented three times, in random order. The paired block consisted of the other four trajectories, presented in the same manner. Within each session, these 8 blocks were presented in random order, in alternation with 9 reference blocks. In these reference blocks (indicated in grey in Table 1), each of the possible target trajectories was presented once, in random order. The two sessions only differed in the random order in which the eight experimental blocks were presented, and the random order in which the trajectories were presented within each block. Except for the authors, participants were not aware that the targets were presented in blocks.

In the accelerating and decelerating blocks (indicated in red in Table 1) the targets were all accelerating or all decelerating, so performance could be improved by aiming for a position that the target will reach slightly later or earlier than the time of the tap (further in front of or behind the target). In the error grouped blocks (indicated in blue) rightward moving targets were decelerating and leftward moving targets were accelerating, or vice versa, so ignoring acceleration would result in systematically tapping to the right (or left) of the target. Therefore, performance could be improved by compensating for these errors by tapping to the left (or to the right) of where one estimated that one should tap on the basis of the target’s position and velocity. In the two remaining pairs of blocks there is no simple way to improve performance without actually considering acceleration. In the speed grouped blocks (indicated in orange), the targets were either all relatively slow or fast (or equivalently all started closer or further from the screen centre). In the direction grouped blocks (indicated in green), the targets either all moved to the right or all moved to the left.

The reference blocks contain all trajectories, so we expect participants to make systematic errors in accordance with ignoring the acceleration during the last part of the movement. The magnitude of the errors can be estimated from the model described by Eqs. 2 and 3 (in which acceleration is not taken into consideration). If the circumstances are such that participants can reduce the magnitude of the errors by for instance tapping earlier or further to the right, participants might gradually do so. Thus, the errors might gradually decrease during the acceleration grouped and error grouped blocks, but not during the other blocks. To help interpret the data in terms of using such heuristics, we consider errors to be positive if they are consistent with ignoring the acceleration. This is the case when hitting ahead of decelerating targets or behind accelerating targets. Defining errors in this manner makes it meaningful to average errors across pairs of blocks as well as sessions to evaluate to what extent extrapolation errors that arise from ignoring acceleration are compensated for by aiming for a different position.

Since the order in which the trajectories were presented was randomized, and we expected participants to only gradually learn to compensate for the extrapolation errors that arise from ignoring acceleration in the blocks in which it was possible to do so, we combined the errors within sets of 4 consecutive trials for all blocks except the reference blocks. For each pair of blocks this gave us three values per participant, each of which is the median of the error on 16 trials: 4 consecutive trials for two selections (e.g., leftward and rightward motion) each presented twice (once in each of the two sessions). For the reference blocks we simply determined the median of all 144 errors per participant. We also determined the error predicted by simulations with the same model and parameters as were used in Experiments 2–4. This included the slow accumulation of velocity information both during and across trials (a rate of updating of 2% per step; Eq. 3), with each trajectory being considered for the number of steps that were shown in a randomly selected trial. There was no random component to the updating of the estimate of velocity within trials, but variability is introduced by the random order in which the blocks were presented and in which the trajectories were presented within the blocks. We simulated the same orders as were used in the actual experiment.

### Results

Of the total of 5712 trials (17 participants each performed 2 sessions of 168 trials), there were 32 in which no tap was detected and 13 with missing data. For the remaining trials, the reaction time and movement time were 239 ± 21 ms and 260 ± 40 ms, respectively. The systematic error in the direction that is consistent with ignoring acceleration was about 4.5 mm in the reference blocks (dashed line in Fig. 10A). As anticipated, participants did not learn to cope with acceleration in the direction grouped blocks (green symbols) or in the speed-grouped block (orange symbols). They did learn to cope with acceleration in the acceleration grouped blocks (red symbols). They also learnt to cope with random alternations of acceleration and deceleration in error grouped blocks (blue symbols). The model described by Eqs. 2 and 3 predicted an average error of 8.2 mm, which is considerably larger than the average error that we found in any of the blocks, and is therefore not plotted in Fig. 10A.

### Discussion

The most important finding of Experiment 5 is that participants learnt to aim further to the left or to the right of targets when they were grouped by error, despite the targets having different accelerations (blue symbols in Fig. 10A). As expected, they also learnt to aim further ahead of or behind targets when they were grouped by acceleration (red symbols in Fig. 10A). This confirms the idea that people use heuristics for dealing with acceleration. Participants learnt to cope with acceleration by adjusting their movements on the basis of recent errors, compensating for systematically tapping too early or too late or too far to one side. To further evaluate whether such heuristics could be responsible for the observed data, we expanded our model to include such heuristics.

#### Modelling these results

Using the same gradual updating of velocity that helped account for the tapping errors in Experiments 2–4 (turquoise curves in Figs. 5C, 6C, 8B and 8D), our model predicts systematic errors of about 8.2 mm. We found much smaller errors than our model predicts, even in the blocks in which we see no evidence that participants learnt to deal with the acceleration (Fig. 10A). In Experiments 2–4 the velocity was quite variable from frame to frame, without changing systematically within or between trials, so it was never disadvantageous to accumulate information about the velocity across long periods of time. In contrast, in Experiment 5 the velocity differed across trials and was constantly changing in a systematic manner within a trial, so it was not advantageous to accumulate information across extended periods of time. Thus, participants may have learnt not to accumulate velocity information so gradually in Experiment 5. However, we cannot with certainty attribute the smaller error than predicted to a change in how information about the target’s velocity is accumulated, because the influence of the initial 100 ms of target motion in Experiments 3 and 4 was also systematically smaller than the model predicts (Figs. 6A and 7). The estimate of target velocity is therefore probably derived in a more complicated manner than is captured by Eq. 3. However, for modelling the results we will assume that velocity information is indeed simply accumulated across less time in Experiment 5.

Reducing the time across which information is accumulated by updating the velocity by 15% rather than 2% at each step gives rise to an average error of about 4.5 mm (as found in the reference blocks of Experiment 4; dashed line in Fig. 10A). We therefore increased the updating rate in Eq. 3 to get

4 $${v}_{s}=0.85 {v}_{s-1}+0.15 {v}_{step}.$$

We incorporated the heuristics that are used to compensate for errors in the model (Eq. 1) by adding two terms, corresponding with the possibility of adjusting the position that participants are aiming for both in time (${C}_{i}^{t}$) and space (${C}_{i}^{s}$). The prediction for the position that the participant is aiming for on trial *i* then becomes:

5 $${A}_{t,i}={T}_{t-\updelta }+{v}_{t-\updelta }\left({t}_{tap}+{C}_{i}^{t}-\left(t-\updelta \right)\right)+{C}_{i}^{s}.$$

In this equation, ${C}_{i}^{s}$ is a spatial correction (further to the left or right) that is updated after each tap on the basis of the finger’s position with respect to the target at the time of the tap. We assume that we slowly forget this correction (van der Kooij et al. 2015), so we let its value decrease to 95% of its former value after each tap. Assuming that the finger arrives at the position that it aims for (${A}_{tap}$) at the anticipated time, the finger’s position with respect to the target is (${{A}_{tap}-T}_{tap}$). The value of ${C}^{s}$ on the next trial *i* + *1* is adjusted to compensate for 25% of this error.

6 $${C}_{i+1}^{s}={0.95 C}_{i}^{s}-0.25 \left({{A}_{tap}-T}_{tap}\right).$$

This is a popular way of simulating error correction (Smith et al. 2006; van Beers 2009; van Beers et al. 2013; van der Kooij et al. 2015). $${C}^{t}$$ is updated in a similar manner after each tap on the basis of the difference in time between when the target passed, or would have passed, the position that was tapped ($${t}_{passed})$$ and the time of the tap. The value of $${C}^{t}$$ on the next trial, and therefore the time used to predict where the target will be at the time of the tap on the next trial, is adjusted to compensate for 15% of the error.

7 $${C}_{i+1}^{t}=0.95 {C}_{i}^{t}-0.15 \left({t}_{passed}-{t}_{tap}\right).$$

With faster updating (Eq. 4) and the additional error-correction (Eqs. 5–7) the model behaves quite similarly to the participants in Experiment 4. Figure 10B shows mean errors of 1000 simulations of the whole experiment, with 17 participants who each take part in 2 sessions (both corrections start at zero at the beginning of each session; $${C}_{0}^{t}=0;{C}_{0}^{s}=0)$$. Again, we did not formally fit any of the parameters, but selected the updating rate for judging velocity such that the overall errors of data and model would match, and selected the percentages by which the two kinds of errors were adjusted after each trial such that the learning rates would more or less match the data.

To account for the improvement in performance both within acceleration grouped blocks of trials (in which it is beneficial to plan to tap earlier or later; red symbols in Fig. 10A) and within error grouped blocks of trials (in which it is beneficial to plan to tap further to one side; blue symbols in Fig. 10A), we had to assume that participants respond to errors by adjusting offsets in both the time and the position that determine where they aim. Both are needed because only adjusting the timing can reduce errors in the acceleration grouped blocks but not the error grouped blocks, and only adjusting the position can reduce errors in the error grouped blocks but not the acceleration grouped blocks. What if both adjustments are effective?

In a previous study targets were always moving to the right, and there were alternating blocks of 12 trials in which the target was either always accelerating or always decelerating (Experiment 2 of Brenner et al. 2016). Under such circumstances the systematic error decreased more rapidly than it did in the acceleration grouped or error grouped conditions of the present study (grey symbols in Fig. 10C; these values are the average errors within the blocks in which participants had to tap on all targets, whereby hitting behind accelerating targets or ahead of decelerating targets was considered a positive error so that performance when targets were accelerating and decelerating could be averaged). Simulating that experiment with Eqs. 4–7 reproduced the data quite well (black symbols in Fig. 10C).

Reducing the values of ${C}_{i}^{t}$ and ${C}_{i}^{s}$ to 95% of the adjusted value after each adjustment was not essential for simulating the data of Experiment 5 (Fig. 10A), but it was essential for simulating the study in which both adjustments helped compensate for the acceleration (Fig. 10C). Without the slow drift toward the value with no correction the simulation was unstable: we obtained simultaneous compensatory drifts of ${C}_{i}^{t}$ and ${C}_{i}^{s}$. The existence of such slow drift back to an original state has been noted before (Smeets et al. 2006; Smith et al. 2006; van der Kooij et al. 2015). The instability that can arise from simultaneously adjusting related measures shows that such drift can be beneficial.

That we could reproduce the way in which participants coped with or failed to cope with acceleration so well with such a simple adaptation of the model supports the idea that people use heuristics for dealing with acceleration. The lateral adjustments that we modeled are probably the same mechanisms that give rise to corrections for spatial offsets in many other circumstances (Redding and Wallace 2003; van den Dobbelsteen et al. 2003). The temporal adjustments are probably the same mechanisms that give rise to corrections for additional delays (de la Malla et al. 2012; Rohde et al. 2014). They are possibly also useful in dealing with small systematic variations in the visuomotor delay as a result of factors such as stimulus contrast (Veerman et al. 2008). Correcting for errors across similar subsequent actions is probably an effective way of dealing with many factors that one cannot judge reliably enough, or respond to quickly enough, for doing so to be effective. Of course, this is only useful when the circumstances do not change, but in daily life we do often repeat actions many times under similar circumstances.

## General discussion

We examined the extent to which visual information about a moving target is accumulated to guide an ongoing interceptive action. We found that people relied on the latest information about position and accumulated information about velocity. A model combining these sources of information with learning from errors could account for our experimental findings.

### Position

In Experiments 1–4, every change in target position resulted in an appropriate response for dealing with that change. The response to each of the many changes was very similar to previously found responses to isolated changes (Brenner and Smeets 1997; Zhang et al. 2018). The vigour of the response was adjusted to the remaining time, and scaled approximately linearly with the step size (until it approached some maximal value that is presumably determined by mechanical constraints; green and blue triangles in Fig. 9).

We see no evidence for accumulation of information in an attempt to obtain a better estimate of the position. In particular, steps 150 ms or longer before the tap were usually completely compensated for (Figs. 3B, 5D, 6C, 8B, D and F). With a latency of 100 ms, the correction had to be made within 50 ms. Since it obviously takes time to execute such a correction (Figs. 2C, 5B, 6B, 8A, C and E), it is evident that information about the target’s position must be used very quickly, rather than gradually being accumulated across tens of milliseconds.

The vigour of the response to each step depended on the remaining time until the tap (Figs. 2C, 3A, 8A, C and E). The vigour is more or less consistent with optimizing smoothness (Minimizing jerk; Fig. 4A), except when the remaining time is short. For all times, it is well described by a mass-spring-damper system that stiffens linearly during the movement (Fig. 4B). A mass-spring-damper system captures some of the neuromechanical properties of muscles and of the inertia of the arm. The time constants that have been reported for such systems are about 100 ms (Soechting et al. 1981; Lemaire et al. 2016). This time constant is consistent with fusion of twitches occurring at typical firing rates of motor neurons (Milner-Brown et al. 1973) and resembles the typical duration of responses in our study (Figs. 2C, 5B, 6B, 8A, C and E). The resulting low-pass filtering will smooth out any high-frequency jitter that arises from constantly using the instantaneous estimate of the position to determine where to aim, while the arm will still always respond to the latest information, which is obviously very useful for dealing with unpredictable motion. Including a model that could capture some of the neuromechanical properties of fast responses (Fig. 4B) lends credibility to our explanation for the observed overshoot in the corrections for steps between 200 and 150 ms before the tap (Figs. 3B, 5D, 6C, 8B, 8D and 8F).

One might argue that relying on the instantaneous position is not a general feature of motor control, but is specifically elicited by our stimulus. For the jitter that we used, relying on the instantaneous position is indeed advantageous, because for the random walk that arises from our random selection of displacements the instantaneous value is the best predictor of future values. We do not think this is the only reason for using instantaneous position information, because we know that people do not avoid responding to target steps, or even decrease the vigour of their responses to such steps, when the target repeatedly steps back (Brenner et al. 2022). They also do not learn to anticipate simple sequences of steps across trials unless they are explicitly aware of the sequence (Oostwoud Wijdenes et al. 2016). Our participants were usually not even aware of the presence of jitter in the rightward moving targets’ velocity in Experiments 2–4, let alone being aware of the nature of the jitter. Nevertheless, the responses to the jitter were very similar for the moving targets of Experiments 2–4 as for the targets that were visibly jittering without moving systematically to the right in Experiment 1. We therefore consider it unlikely that our participants quickly learnt how to best respond to this particular kind of jitter during the experiment. It is more likely that this is generally a good strategy in daily life, as explained in the discussion of Experiment 1. Of course, this does not mean that there can be no situations in which it is advantageous to accumulate information about a target’s position across time. When an experiment is intentionally designed to make it difficult to judge a static target’s instantaneous position, people accumulate information about the target’s position before starting to move towards it (Battaglia and Schrater 2007), so maybe in such situations further updated estimates of the target’s position are used to guide ongoing movements.

### Velocity

The systematic influence that the velocity during the first 100 ms of target motion had on the tapping errors in Experiments 3 and 4 (Figs. 6A and 7) provides direct evidence that people rely on accumulated velocity information to guide their finger when performing an interception task. Participants tapped about 3 mm further along the target’s path when the target initially moved faster, although there was no systematic difference between the target trajectories after the first 100 ms, and the reaction times and movement times were also similar. That the target’s velocity long before the tap influenced the errors is also evident from the grey points on the left of Figs. 5D, 6C, 8B, 8D and 8F all being above zero. The fact that the speed at the end of the previous trial also influenced performance (Fig. 5C) shows that even the velocity of previous targets contributes to the accumulated velocity information. This is in line with previous findings (de Lussanet et al. 2001). Acquiring velocity information obviously takes some time, but the good match between our model (Eqs. 2 and 3; turquoise curves in various figures) and the participants’ data in Experiments 2–4 shows that the accumulation extends across many steps throughout the target’s movement and even across movements, providing a unified explanation for the accumulation within and between trials.

We had to reduce the accumulation substantially (by increasing the rate at which information was updated from 2 to 15% per step) for our model to fit the data of Experiment 5. Comparing the observed influence of the previous trial in Experiment 2 with that in a study in which targets moved at various constant velocities (de Lussanet et al. 2001) supports the idea that the rate at which velocity information is updated depends on the circumstances: in that study the differences between target velocities on previous trials was much larger than in Experiment 2 of the present study, but their influence on the errors was not. This supports the idea that the slow updating of velocity information that we inferred from Experiments 2–4, including considerable accumulation of information across trials, may be specific to targets that have about the same velocity both throughout and across trials. However, when trials always started with 100 ms of either fast or slow motion (Experiments 3 and 4), the rate of accumulation did not decrease sufficiently to prevent such initial motion from giving rise to systematic errors (Figs. 6A and 7).

### Acceleration

We interpret our results as support for the existing evidence that people cannot use visual information about a target’s acceleration to guide their movements (Benguigui and Bennett 2010; Brenner and Smeets 2015; Brenner et al. 2016; Lee et al. 1997, 1983; Port et al. 1997). This is in line with the poor ability to judge acceleration (Brouwer et al. 2002; Calderone and Kaiser 1989; Gottsdanker et al. 1961; Watamaniuk and Heinen 2003; Werkhoven et al. 1992). Acceleration can be considered to some extent under some circumstances, such as when a target is hidden from view after moving for hundreds of milliseconds (Bennett et al. 2007). Presumably, under such circumstances the fact that the target was accelerating or decelerating is inferred from observed changes in velocity (Gottsdanker et al. 1961; Brouwer et al. 2002). If the participants of Experiment 5 had been able to infer whether targets were accelerating or decelerating in this manner, they should have been able to compensate for the acceleration in all blocks, because the magnitude of the acceleration or deceleration was constant throughout the experiment. This was clearly not the case. Moreover, when targets initially moved particularly slowly in Experiments 3 and 4, participants hit further behind the target, in accordance with accumulating information about the velocity (averaging following Eqs. 3 and 4), rather than hitting ahead of the target in accordance with interpreting the increase in target velocity after the first 100 ms as evidence that the target was accelerating.

### Heuristics

It is not farfetched to assume that people learn to remove errors when intercepting moving targets by adjusting where they aim (Eq. 5). Generally, when making goal-directed movements, people quickly learn to cope with imposed systematic spatial offsets and with unusual forces on the moving arm (both reviewed in Fleury et al. 2019), as well as with modest time delays (de la Malla et al. 2012). It has also been shown that adaptation to systematic spatial offsets is incomplete, even after extensive exposure (van der Kooij et al. 2015). Moreover, the adaptation gradually disappears when there is no feedback, even if this consists of drifting away from a correct match (Smeets et al. 2006; van der Kooij et al. 2013). Why do we quickly forget most of what we learn?

When modelling the results of Experiment 5, we had to consider two different ways to cope with observed errors (Eqs. 5–7). In an attempt to keep the model as simple as possible, we initially modelled the data without forgetting. However, we discovered that when simulating a situation in which both ways of coping with the error were effective (Fig. 10C), we had to add gradual drift back to the original state to prevent complementary adjustments in the two learnt values (${C}_{i}^{t}$ and ${C}_{i}^{s}$) from compensating for each other such that both the values shift away from the ‘correct’ value together. We propose that the observed drift back to the natural state (Smeets et al. 2006; van der Kooij et al. 2013) is therefore not just a limitation of learning, but that such ‘forgetting’ is useful whenever there are multiple options for compensating for errors, which is probably often the case.

### Stages in goal-directed movements

In 1899, Woodsworth proposed that reaching movements consist of two phases: an initial ballistic phase that reflects the initial plan and a later slow phase that is controlled through feedback loops. Although there is a lot of evidence that the whole movement is controlled (Brenner and Smeets 2018; Smeets and Brenner, 1995b), some version of this idea is still quite prominent in theories about goal-directed movements (reviewed in Elliott et al. 2017). The current study provides an explanation for why scientists may often interpret experiments in that way: the beginning of a movement looks somewhat ballistic because adjustments are not very vigorous (Fig. 3A). However, the adjustments are just as adequate. Responses only become more vigorous when the target is displaced later during the movement because the adjustments have to be made within less time (Figs. 2C, 5B, 6B, 8A, 8C and 8E; Brenner et al. 2022; Oostwoud Wijdenes et al. 2011; Zhang et al. 2018).

Having less time until the end of the movement is not the only reason to adjust response vigour. Responses also become more vigorous when the urgency of the correction is increased in other ways (Crevecoeur et al. 2013; Franklin and Wolpert, 2008; Keyser et al. 2017, 2019; Knill et al. 2011) or if the task requires a stronger response for other reasons (Franklin et al. 2017). Changes in urgency can therefore provide a unifying explanation for the claims that information is used fundamentally differently at different stages of a movement (Elliott et al. 2017) and that the optimal time for responding to perturbations depends on factors such as the arm’s velocity (Tremblay et al. 2017). Based on our results, we conclude that responses are based on the same information and have the same latency during the whole movement. The difference between responses at different times is that the vigour of the response depends on the remaining time (Brenner et al. 2022). Adjustments are more vigorous later in the movement, because the adjustment needs to be made within less time.

### How movements are controlled

Our reasoning about how visual information is used to guide goal-directed movements combines ideas from two theories about optimizing movement control. The first is that the combination of visual information and movement pattern is used that minimizes the anticipated errors due to random variability in sensorimotor signals (Harris and Wolpert 1998). The second is that movements are constantly adjusted to reach their goal, rather than being adjusted to follow an initial plan (optimal feedback control; Scott 2004; Todorov 2004; Todorov and Jordan 2002). Considering that visual estimates of positions are quite precise and that egocentric positions change whenever an object or the observer moves, using the latest position might be optimal. Visually estimating velocity takes time, velocity judgments are not very precise, and objects often move at about the same speed for some time. It might therefore be advantageous to accumulate velocity information. Whether it is really advantageous to do so across hundreds of milliseconds remains to be seen, but we found some evidence that the duration of accumulation may depend on the circumstances. The duration might even constantly be updated on the basis of systematic errors in predicting where the target will be, in a similar manner to the way in which the aiming point is adjusted on the basis of tapping errors (Eqs. 6 and 7).

**Fig. 11:**
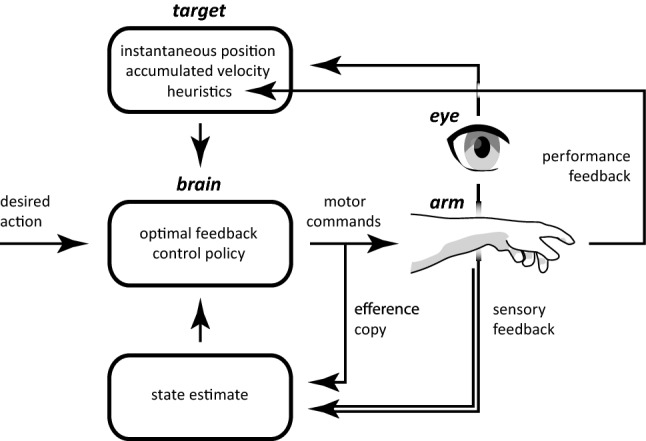
Extension of the optimal feedback control model (Scott 2004) to include information about the outside world. For our task, sensory information about the target is combined with heuristics based on feedback from recent attempts as well as the estimated state of the arm to determine the optimal feedback control policy for guiding the arm towards the target

The present study examines how visual information about the target is used to guide goal-directed actions. Our main question is how people determine the point towards which the arm is guided at each moment. This complements the many studies that are concerned with subsequently guiding the arm to this point (Diedrichsen et al. 2010; Scott, 2004, 2012), including ones concerned with how visual information about the moving arm contributes to such guidance (Cámara et al. 2018; Yeo et al. 2016) or fails to do so (Crevecoeur et al. 2016). A target’s motion is usually less predictable than that of one’s own arm. Moreover, when guiding the arm to a certain point one could benefit from efferent signals to overcome some of the sensorimotor delays that are encountered when dealing with visual or haptic feedback about the arm (Desmurget and Grafton 2000; Pickering and Clark 2014). When using visual information about the target to determine where to guide the arm, one must predict where the target will be at some time in the future from visual information alone. We examined how such visual information is used. A schematic view of how such information could be incorporated within the *optimal feedback control model* to consider estimates about the outside world (in our case the target) in addition to estimates of the state of (the arm of) the actor is shown in Fig. 11.

In this study, we only consider simple goal-directed movements in which the only thing that matters is reaching the target at the time of the tap. Obviously, the way in which the movement is planned and controlled also depends on any subsequent actions (Rosenbaum and Sauerberger 2019), additional requirements such as having to hit the target in a certain direction or having to avoid hitting obstacles (Brenner and Smeets 2007), and sometimes even wanting to move in a certain manner because the way one moves is relevant to the task, as is often the case when dancing or making music (James 2018).

## Conclusion

Overall, our study shows that people rely heavily on the latest visual estimate of the target’s *position* to guide their ongoing movements. They rely on a visual estimate of its *motion* to extrapolate this position to the anticipated moment of contact. This motion estimate is gradually updated as the movement unfolds, and therefore reflects velocity information accumulated over hundreds of milliseconds. By adjusting subsequent movements to compensate for any errors, people consider persistent target acceleration, as well as systematic errors that arise from any other factors such as optical deformation, muscle fatigue, or the presence of external forces. Apparently, this combination of continuous control based on limited information and quickly learning from previous errors when confronted with similar conditions on subsequent trials can give rise to the amazing performance that is observed in human interactions with objects under demanding circumstances such as one encounters in various sports and in daily life when reaching to grasp small objects.

## Data Availability

The datasets and analysis scripts of the current study are available at https://osf.io/y8x42/?view_only=3a927c596a9747ee9da1d06ab47265a6.
